# Interactome of intact chromatosome variants with site-specifically ubiquitylated and acetylated linker histone H1.2

**DOI:** 10.1093/nar/gkad1113

**Published:** 2023-11-22

**Authors:** Philip Saumer, Martin Scheffner, Andreas Marx, Florian Stengel

**Affiliations:** Department of Chemistry, University of Konstanz; Universitätsstraße 10, 78464 Konstanz, Germany; Konstanz Research School Chemical Biology, University of Konstanz; Universitätsstraße 10, 78464 Konstanz, Germany; Konstanz Research School Chemical Biology, University of Konstanz; Universitätsstraße 10, 78464 Konstanz, Germany; Department of Biology, University of Konstanz; Universitätsstraße 10, 78464 Konstanz, Germany; Department of Chemistry, University of Konstanz; Universitätsstraße 10, 78464 Konstanz, Germany; Konstanz Research School Chemical Biology, University of Konstanz; Universitätsstraße 10, 78464 Konstanz, Germany; Konstanz Research School Chemical Biology, University of Konstanz; Universitätsstraße 10, 78464 Konstanz, Germany; Department of Biology, University of Konstanz; Universitätsstraße 10, 78464 Konstanz, Germany

## Abstract

Post-translational modifications (PTMs) of histones have fundamental effects on chromatin structure and function. While the impact of PTMs on the function of core histones are increasingly well understood, this is much less the case for modifications of linker histone H1, which is at least in part due to a lack of proper tools. In this work, we establish the assembly of intact chromatosomes containing site-specifically ubiquitylated and acetylated linker histone H1.2 variants obtained by a combination of chemical biology approaches. We then use these complexes in a tailored affinity enrichment mass spectrometry workflow to identify and comprehensively characterize chromatosome-specific cellular interactomes and the impact of site-specific linker histone modifications on a proteome-wide scale. We validate and benchmark our approach by western-blotting and by confirming the involvement of chromatin-bound H1.2 in the recruitment of proteins involved in DNA double-strand break repair using an *in vitro* ligation assay. We relate our data to previous work and in particular compare it to data on modification-specific interaction partners of free H1. Taken together, our data supports the role of chromatin-bound H1 as a regulatory protein with distinct functions beyond DNA compaction and constitutes an important resource for future investigations of histone epigenetic modifications.

## Graphical Abstract

**Graphical Abstract:**
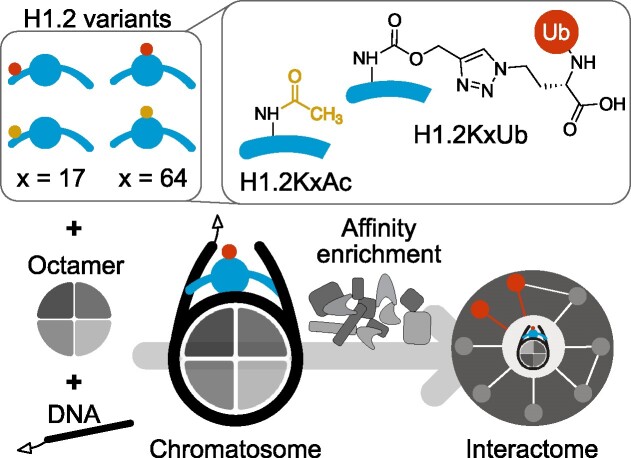

## Introduction

In eukaryotes, DNA is stored in the nucleus in the form of chromatin. Compaction of the DNA is achieved by its association with an octamer of core histone proteins H2A, H2B, H3 and H4 to form nucleosomes. Binding of linker histone H1 to nucleosomes results in further compaction and the formation of chromatosomes (1). Various post-translational modifications (PTMs), that are predominantly found on the tails of core histones, then mediate access to DNA for transcription and DNA replication (2,3). Linker histones are also diversely modified and while there is growing evidence that these modifications are as well involved in the regulation of chromatin-based processes, their actual function(s) remain relatively poorly characterized (4–8).

Affinity-enrichment mass spectrometry (AE-MS) is an established method to investigate protein-protein interactions (PPIs) from complex mixtures like cell lysates with the help of an affinity tag attached to the bait molecule (9). Enriched interactors are subsequently identified and quantified by LC-MS/MS revealing bait-specific interactions. Chromatin components or parts thereof, like histone peptides, histone proteins, or recombinantly prepared nucleosomes, carrying defined modifications introduced by chemical biology approaches, have already been used successfully as bait molecules in AE-MS experiments (10–18). Yet, investigations on the function of histone PTMs have been largely focused on core histone PTMs, while only recently insights into the interactomes of site-specifically modified H1 variants on their own were obtained (14,15).

To elucidate the specific influence of PTMs on chromatin, complex structures that closely resemble native chromatin are preferable bait molecules, since only they allow multivalent interactions that likely occur on chromatin (19,20). We assembled intact chromatosome variants by combination of different site-specifically modified linker histone variants generated by bioorthogonal chemistry with recombinantly expressed core histones and nucleosomal DNA that carried an affinity tag. Their application as baits in AE-MS experiments allows for the first time a direct comparison of nucleosome- and chromatosome specific interaction partners as well as obtaining insights into the effect of site-specific PTMs on histone H1.2.

Our data reveals modification-dependent interaction partners of chromatin-bound H1.2 and provides evidence for a role of chromatin-associated H1.2 in the recruitment of proteins involved in DNA double-strand break repair. Overall, it supports the role of H1.2 as a regulating protein with distinct functions beyond DNA compaction and constitutes an important resource for future investigations of histone epigenetic modifications.

## Materials and methods

### Materials

Unless specified otherwise, ultrapure water was used as obtained from a Milli-Q purification system (Merck). All chemicals were purchased from Sigma Aldrich/Merck, VWR, Carl Roth, abcr, Acros organics, TCI or Fluka in analytical or molecular biology grade unless stated otherwise. Chemicals and solvents used for mass spectrometric analyses were purchased in LC-MS grade or UHPLC-MS grade. Oligonucleotides for the PCR of nucleosomal DNA were purchased from biomers.net. The reverse primer was purchased HPLC purified and carrying a 5′-desthiobiotin modification, the forward primer was purchased without modifications and RP-cartridge purified. Oligonucleotides for the double strand DNA ligation assay were purchased from IDT with PAGE purification by the manufacturer. Oligonucleotide sequences were as indicated in Supplementary Table S1.

### Expression and purification of core histones

Canonical human core histones H2A and H3 were expressed and purified essentially as described previously (21,22). Briefly, histones were expressed from pET-11a vectors in *Escherichia coli* BL21 (DE3) and purified by anion exchange filtering (HiTrap Q HP column, Cytiva) followed by cation exchange purification (HiTrap SP HP column, Cytiva) under denaturing conditions. Core histones H2B and H4 were expressed with an N-terminal His_6_-tag and TEV cleavage site from pET-15b vectors in *E. coli* BL21 (DE3). For H4 a codon-optimized sequence was used (23). Both H2B and H4 were first purified under denaturing conditions from isolated inclusion bodies via affinity chromatography using cOmplete His-Tag Purification Resin (Roche). After TEV cleavage, H2B was purified by additional affinity chromatography (HisTrap HP column, Cytiva) and H4 was purified by additional cation exchange chromatography (HiTrap SP HP column, Cytiva), both under denaturing conditions.

Purified H2A, H2B, H3 and H4 were extensively dialyzed against water at 4°C and stored flash frozen at -80°C. Protein concentrations were determined by measuring the absorption at 280 nm by NanoDrop (Thermo Fisher Scientific) using extinction coefficients and molecular weight as calculated with the ExPASy ProtParam tool (24) from the amino acid sequences given in Supplementary Table S2.

### Assembly and purification of the core histone octamer

The core histone octamer was prepared as described previously (21). Lyophilized core histones were resuspended in unfolding buffer (20 mM Tris–HCl p H7.5, 5 mM DTT, 7 M guanidine–HCl) and mixed with a 1.2-fold molar excess of H2A and H2B over H3 and H4 to ensure the formation of histone octamer and a H2A–H2B dimer, which is subsequently easily removed by size exclusion chromatography (22). After dialysis against refolding buffer (10 mM Tris–HCl pH 7.5, 2 M NaCl, 1 mM EDTA, 5 mM β-mercaptoethanol) and subsequent concentration by ultrafiltration (10 000 MWCO Amicon, Merck) the histone octamer was purified by size exclusion chromatography (HiLoad 16/600 200 pg column, Cytiva) in refolding buffer. Core histone octamer was flash frozen in liquid nitrogen and stored at –80°C after addition of 10% glycerol.

### Preparation of nucleosomal DNA

The DNA used for nucleosome assembly was based on a published 207 bp sequence that contains a 601-based nucleosome positioning sequence (25) as well as 30 bp of adjacent linker DNA that showed excellent binding of linker histones to nucleosomes (26). After cloning into a pUC-18 backbone, nucleosomal DNA was amplified by PCR in large scale in a 96-well plate with 50 μl reaction size using primers indicated in Supplementary Table S1. The final concentrations in the PCR mixture were 1× Thermopol buffer (NEB), 0.02 ng/μl template DNA, 0.2 mM of each dNTP, 0.25 mM of each primer and 0.025 U/μl of Taq DNA polymerase (NEB). After initial denaturation for 60 s at 95°C, 30 cycles of denaturation at 95°C (15 s), annealing at 48.3°C (20 s) and extension at 72°C (20 s) were performed followed by a final extension at 72°C (5 min). The reverse primer contained a 5′ desthiobiotin (DTB) modification followed by a 10 deoxythymidine-spacer to allow immobilization on streptavidin beads with increased distance between bead surface and chromatosomes to provide space for the binding of interactors. The DNA sequence after PCR with the desthiobiotin-modified primer was:

5′-*DTB*-TTTTTTTTTTTATCTAATACTAGGACCCTATACGCGGCCGCATCGGAGAATCCCGGTGCCGAGGCCGCTCAATTGGTCGTAGACAGCTCTAGCACCGCTTAAACGCACGTACGCGCTGTCCCCCGCGTTTTAACCGCCAAGGGGATTACTCCCTAGTCTCCAGGCACGTGTCAGATATATACATCGATTGCATGTGGATCCGAATTCATATTAATGAT-3′.

After PCR, the DNA was precipitated with –20°C 2-propanol, the resulting pellet was washed with –20°C 95% ethanol and resuspended in water. DNA concentration was determined by measuring the absorption at 260 nm using NanoDrop (Thermo Fisher Scientific).

### Nucleosome assembly

Nucleosomes were assembled by stepwise salt dialysis as described (21,27). The optimal ratio of core histone octamer to DTB-labeled nucleosomal DNA ensuring complete conversion of all DNA into nucleosomes was determined in small scale titrations and kept constant for all following experiments. DTB-DNA (1.0 eq.) was supplemented with a competitor DNA (crDNA; 0.005 eq.; empty pUC-18 vector), BSA (3.85 eq.; Thermo Fisher Scientific) and histone octamer as determined previously. Dialysis in 3500 MWCO tubing (Thermo Fisher Scientific) was started at 4°C in high salt buffer (10 mM Tris–HCl, pH 7.5, 2 M NaCl, 1 mM EDTA, 0.05% Nonidet *P*-40 substitute (v/v), 1 mM β-mercaptoethanol) and the salt concentration was lowered to a final concentration of 50 mM NaCl over the course of 20 hrs by stepwise addition of low salt buffer (same as high salt buffer, but only 50 mM NaCl). All nucleosome assemblies were monitored by 7.5% native PAGE in 0.5x TBE buffer and visualization of DNA by UV irradiation after ethidium bromide staining.

Nucleosome concentrations were determined by measuring DNA concentration by measuring the absorption at 260 nm using NanoDrop (Thermo Fisher Scientific). The obtained DNA concentration was converted to the molar nucleosome concentration using the molecular weight of the nucleosomal DNA.

### Expression and purification of H1.2, H1.2KxPlk and H1.2KxAc

Linker histone H1.2, H1.2KxPlk (15) or H1.2KxAc (14) were expressed and purified as described with minor changes. Briefly, to introduce propargyl-derivatized l-lysine (Plk) or N_ϵ_-acetyl-l-lysine (AcK), H1.2 constructs were used, where the codon for lysine 17 or 64 had been replaced with an amber stop codon (TAG/UAG). All H1.2 variants within this study were expressed with a C-terminal His_6_-tag from pET-11a vectors that contained a second expression cassette for tRNA^Pyl^ needed for amber stop codon suppression. *Escherichia coli* BL21 (DE3) cells were transformed with the respective H1.2 plasmid as well as a pRSF-Duet1 vector containing the particular amino acyl-tRNA synthetase (aaRS) needed for incorporation of Plk or AcK. Transformed cells were incubated at 37°C. At an OD_600_ of 0.3–0.4 either Plk (synthesized as described (28), final concentration 2 mM) or AcK (abcr, final concentration 10 mM) and nicotinamide (Sigma-Aldrich, final concentration 20 mM) were added. For the expression of unmodified H1.2 neither Plk nor AcK or nicotinamide were added, and the aaRS co-transformation step was omitted. Expression was induced by addition of 1 mM IPTG at an OD_600_ of 0.6-0.8 and cells were harvested by centrifugation after 3–5 h at 37°C. Isolation of H1.2 from inclusion bodies and purification via affinity chromatography using cOmplete His-Tag Purification Resin (Roche) was performed under denaturing conditions. Pure proteins were dialyzed against water and stored flash frozen at –80°C. Protein concentration was determined by BCA assay (Thermo Fisher Scientific) or SDS-PAGE and Coomassie staining.

### Preparation of H1.2KxUb by Cu(I)-catalyzed azide-alkyne cycloaddition (CuAAC, click reaction)

Azide-modified Ubiquitin (UbG76Aha) was prepared as described earlier by introducing azidohomoalanine (Aha) at the C terminus through selective pressure incorporation (15,29). The CuAAC between azide modified ubiquitin and alkyne modified H1.2 was performed as described (15). Briefly, H1.2KxPlk (1.0 eq., 10 μM) was combined with UbG76Aha (1.5 eq., 15 μM), THPTA (10 mM), Cu(MeCN)_4_BF_4_ (5 mM) and SDS (K17: 0.8 mM, K64: 0.3 mM) in either 1× PBS (K17) or 20 mM Tris pH 7.5 (K64) under argon atmosphere on ice. After 1 h the reaction was stopped by addition of 50 mM EDTA. Reaction monitoring by SDS-PAGE showed excellent conversion of H1.2KxPlk leaving almost no residual starting material. Aggregates of ubiquitylated H1.2 that formed during the reaction were pelleted by centrifugation. The reaction supernatant was washed with 20 mM Tris pH 7.5, 300 mM NaCl and 20 mM Tris pH 7.5, 500 mM NaCl several times by ultrafiltration (10′000 MWCO, Vivaspin). The pellet was refolded by dialysis under denaturing conditions (50 mM Tris pH 7.0, 1 M NaCl, 6 M urea, 10 mM β-mercaptoethanol) followed by stepwise removal of urea by addition of water and final dialysis against water. H1.2KxUb from pellet and supernatant were combined, further concentrated by ultrafiltration, and stored flash frozen at –20°C. Protein concentrations were determined by SDS-PAGE and Coomassie staining.

### Chromatosome assembly

Nucleosomes containing DTB-DNA were combined with H1.2, H1.2KxUb or H1.2KxAc in low salt buffer (10 mM Tris–HCl pH 7.5, 50 mM NaCl, 1 mM EDTA, 0.05% Nonidet *P*-40 substitute (v/v), 1 mM β-mercaptoethanol) and the mixture was incubated at 19.5°C for 35 min. Success of the chromatosome assembly was verified by 7.5% native PAGE in 0.5× TBE buffer with visualization of DNA by UV irradiation after ethidium bromide staining. Small-scale titration reactions were performed to establish the optimal ratio of H1.2, H1.2KxUb or H1.2KxAc to nucleosomes. The ratio was chosen high enough to ensure complete conversion of all nucleosomes into chromatosomes and low enough that there was no aggregation visible in native PAGE. Due to inevitable variances in concentration determination of all components the optimal molar ratio of H1.2: nucleosome varied from 0.6:1 to 1.1:1 for the different H1.2 variants used. For large scale reactions, the established ratios were kept constant.

### HEK 293T cell culture and lysate preparation

HEK 293T cells were cultured in Dulbecco's modified Eagle medium (gibco) supplemented with 10% (v/v) fetal bovine serum and 1% (v/v) Penicillin/Streptomycin at 37°C and 5% CO_2_. Cells were harvested by scraping, washed with ice-cold 1× PBS and flash-frozen pellets were stored at –80°C.

To prepare lysate, pellets were resuspended in lysis buffer (10 mM Tris–HCl pH 7.5, 50 mM NaCl, 1 mM EDTA, 0.05% Nonidet *P*-40 substitute (v/v), 1 mM β-mercaptoethanol, 2 mM MgCl_2_, 1× Roche cOmplete EDTA-free protease inhibitors) and incubated for 10 min on ice. After lysis by sonication, the lysate was cleared by centrifugation (30 min, 15 000 g, 4°C) and protein concentration of the supernatant was determined by Bradford assay (Roti-Quant, Carl Roth).

### Affinity enrichment of chromatosome interactors

One set of affinity enrichment experiments consisted of seven samples: chromatosome with unmodified H1.2, H1.2K17Ub, H1.2K64Ub, H1.2K17Ac or H1.2K64Ac, respectively as well as nucleosomes and empty beads as control. All steps were performed in low binding tubes (Biozym). Magnetic Dynabeads MyOne Streptavidin T1 beads (Thermo Fisher Scientific) were equilibrated with low salt buffer (10 mM Tris–HCl pH 7.5, 50 mM NaCl, 1 mM EDTA, 0.05% Nonidet *P*-40 substitute (v/v), 1 mM β-mercaptoethanol) and for one set of experiments 90 pmol of nucleosome and each chromatosome variant were immobilized on beads by incubation overnight at 4°C with gentle agitation in a total volume of 180 μl. For the empty bead control, beads were incubated only with low salt buffer. Complete immobilization was verified by probing the supernatant after incubation via native PAGE. After washing the beads with low salt buffer to remove residual BSA and crDNA from nucleosome and chromatosome assembly, HEK 293T cell lysate was added to the beads to a final concentration of 5 mg/ml in a total volume of 180 μl followed by incubation for 2 h at 4°C under gentle agitation. To remove unspecific binding proteins, the beads were washed with low salt buffer, followed by elution of bound proteins with elution buffer (0.8 mM biotin in low salt buffer without Nonidet *P*-40 substitute). Elution was performed five times, each time resuspending the beads in elution buffer followed by incubation at 37°C for 10 min with shaking. Combined elution fractions were passed over HiPPR detergent removal spin columns (Thermo Fisher Scientific) to remove residual detergent. Detergent-free samples were flash-frozen and lyophilized. The affinity enrichment procedure was performed in biological triplicates.

### Chromatosome integrity assay in HEK 293T lysate

Immobilization of chromatosomes was performed as described above. Immobilized nucleosomes were incubated with HEK 293T cell lysate at a final concentration of 5 mg/ml at 4°C under gentle agitation. At different time points a part of the bead mixture was removed, washed with low salt buffer and elution of chromatosomes was performed as described above. The sample for the time point ‘0 h’ was removed before addition of HEK 293T cell lysate. Eluted chromatosomes were analyzed by 7.5% native PAGE in 0.5× TBE buffer with visualization of DNA by UV irradiation after ethidium bromide staining.

### Sample preparation for MS measurements

Lyophilized elution fractions of affinity enrichment procedure were subjected to a tryptic in-solution digest. First, samples were resuspended in urea (8 M) followed by reduction with TCEP (5 mM, 30 min, 37°C with shaking) and alkylation of cysteines with 2-chloroacetamide (10 mM, 30 min, room temperature). After dilution to 1 M urea with 50 mM NH_4_HCO_3_, 2.5 μg sequencing grade trypsin (Promega) was added. The mixture was incubated overnight at 37°C with gentle shaking before the digestion was stopped by addition of 10 % TFA followed by lyophilization. Before LC–MS/MS measurements, the samples were resuspended in 0.1% TFA, acidified with 10% TFA and desalted using μC18 Zip Tips (Merck).

### MS measurement of full-length proteins

Measurements of full-length core histones were performed on a micrOTOF II mass spectrometer (Bruker) coupled to an Agilent 1200 HPLC system. CompassDataAnalysis 4.1 (Bruker) software was used for re-calibration and spectrum deconvolution using the maximum entropy algorithm and default settings.

Measurements of full-length linker histone variants were performed on a 6546 QTOF mass spectrometer (Agilent) coupled to an Infinity II 1260 system (Agilent). MassHunter BioConfirm 10 (Agilent) software was used for spectrum deconvolution using the maximum entropy algorithm. The deconvolution mass range was set to 20 000–40 000 Da, otherwise default settings were used.

### MS measurement of affinity-enrichment samples

Desalted samples from the affinity enrichment step were analyzed on a UHPLC-MS system consisting of an EASY-nLC 1200 UHPLC system (Thermo Fisher Scientific) equipped with a 50 μm x 15 cm C18 Acclaim PepMap RSLC column with 2 μm beads and 100 Å pore size (Thermo Fisher Scientific) coupled to a Q-Exactive HF mass spectrometer (Thermo Fisher Scientific). Peptides were separated over a total gradient length of 120 min: after 4 min at 5% acetonitrile (ACN) the concentration was increased to 35% ACN over 100 min, then to 45% ACN over 5 min followed by a washing step at 80% ACN. Samples were measured in technical duplicates in data independent acquisition (DIA) mode with 22 isolation windows of variable window size. The isolation width and window position are indicated in Supplementary Table S3 and were derived from earlier optimization efforts (30). MS1 scans were acquired from 300 to 1650 *mz* with a resolution of 120 000 (at 200 m/z), a maximum injection time of 60 ms and an AGC target of 3e6. After one MS1 scan, the mass spectrometer cycled once through the indicated 22 DIA windows. Isolated ions were fragmented by HCD with stepped normalized collision energies of 25.5, 27 and 30 eV and fragment ion spectra were acquired with automatic injection time at a resolution of 30 000 (at 200 *mz*) and with an AGC target of 3e6. Spectra were recorded from a fixed first mass of 200 *mz* and with a default charge state of 4. QCloud has been used to monitor the instrument performance during the measurements (31).

### Affinity enrichment: data analysis and identification of interactomes

Raw data from MS measurements were converted to htrms format using HTRMS converter V16 (Biognosis) (32) and default settings. Identification and label-free quantification of proteins was performed using Spectronaut V16 (Biognosys) (32) in library-free directDIA mode. Default settings were used, only the minimum peptide length was adjusted to 5. As protein database, ‘uniprot_sprot_2021–01-04_HUMAN’ as downloaded from the built-in ‘Biognosys Protein Databases Online Repository’ was used.

Further statistical analysis of raw LFQ data was performed with Perseus 1.6.15.0 (33). Since Spectronaut offers no built-in contaminant database, identified potential contaminants were annotated based on an established contaminant database (34) and filtered from the list of identified protein groups. Affinity enrichment was performed in triplicates and samples were measured in technical duplicates resulting in a total of six measurements for each sample. LFQ values were log_2_(x) transformed and only proteins with 4 of 6 valid values in at least one sample were kept. For the remaining 1719 protein groups, missing values were assumed to result from protein groups being below the limit of detection and imputation was performed separately for each measurement from a normal distribution (width = 0.3, downshift = 1.8). The mean LFQ value of technical replicates was calculated and significantly bait enriched proteins were identified by ANOVA (*S*_0_ = 0.1, FDR = 0.05) followed by normalization via *z*-scoring and hierarchical clustering of sample mean *z*-scores according to their Euclidean distance. Clustering revealed two clusters: proteins enriched for either one of the bait samples (nucleosome or one of the chromatosome variants) or for the empty bead control. Continuing only with the bait-enriched cluster consisting of 728 protein groups, pairwise comparisons of LFQ values to the nucleosome sample were performed in order to identify differences resulting from the presence of (un-) modified H1.2. After performing sequential *t*-tests (*S*_0_ = 0.1, FDR = 0.05), 119 proteins remained that showed a significantly changed enrichment in at least one of the pairwise comparisons. Again, *z*-scoring of LFQ values of these protein groups was performed, this time without the LFQ values of the empty bead control. Sample mean *z*-scores were clustered by their Euclidean distance and revealed again two clusters: one consisting of nucleosome enriched proteins groups and one of chromatosome enriched protein groups with differences between the various H1.2 variants. Analysis of enriched GO-terms was performed with PANTHER (35) and was focused on the term ‘protein class’. Further enriched GO-terms were identified with Fisher's exact test (FDR = 0.02) comparing the clusters of protein groups (overall 119 protein groups) that were significantly enriched in pairwise comparisons to all bait enriched protein groups as indicated by ANOVA (728 protein groups).

### Data availability

The mass spectrometry proteomics data have been deposited to the ProteomeXchange Consortium via the PRIDE (36) partner repository with the dataset identifier PXD040236.

### Immunoblotting

For the analysis of protein enrichment by immunoblotting, all assembly and affinity enrichment steps were performed as described above. Elution of enriched proteins was performed directly with SDS-PAGE loading buffer and analysis of proteins was performed by SDS-PAGE followed by western blotting to 0.2 μm PVDF membrane (Amersham, GE). Primary antibodies were directed against DCUND1/2 (dilution 1:500, Santa Cruz sc-398218), POLB (1:500, Santa Cruz sc-376581), PRKDC (1:1000, Cell Signaling Technologies #38168), XRCC4 (1:1′000, Cell Signaling Technologies #23908) and XRCC6 (1:5′000, Proteintech 6607-1-Ig). Proteins were visualized with ECL substrate (Pierce) or SuperSignal substrate (Pierce) after incubation with the corresponding HRP-conjugated secondary antibody.

### Double strand DNA ligation assay

The double-stranded oligonucleotide ligation assay with ^32^P-labeled DNA substrate was performed as described earlier with minor adaptations (37,38). 75mer and 79mer oligonucleotides were purchased as described, their sequences are stated in Supplementary Table S1. The 79mer oligonucleotide was 5′-labeled with γ-^32^P-ATP (Hartmann Analytic). The labeling reaction was performed in a total volume of 20 μl with a final oligonucleotide concentration of 1 μM, 8 U of T4 polynucleotide kinase (T4 PNK, NEB) and 1× T4 PNK reaction buffer (NEB). After incubation at 37°C for 1 h, the reaction was stopped by heating to 95°C for 2 min. Excess γ-^32^P-ATP was removed by gel filtration using Sephadex G-10 resin (Cytiva). Annealing was performed by mixing 5′-labeled 79mer oligonucleotide with an equimolar amount of unlabeled 75mer oligonucleotide in 10 mM Tris pH 7.5 followed by heating to 90°C for 5 min and slow cooling to room temperature.

The DSB assay master mix consisted of 1× end joining buffer (50 mM Tris pH 7.5, 5 mM MgCl_2_, 1 mM ATP, 1 mM DTT, 5% PEG 8000), 1× Roche cOmplete EDTA-free protease inhibitors and 10 ng of ^32^P-labeled DSB substrate per reaction. Assembly of nucleosomes and chromatosomes as well as affinity enrichment were performed as described above. After incubation with HEK 293T cell lysate, beads were washed with low salt buffer (10 mM Tris–HCl pH 7.5, 50 mM NaCl, 1 mM EDTA, 0.05% Nonidet *P*-40 substitute (v/v), 1 mM β-mercaptoethanol), resuspended in 2 μl of low salt buffer and supplemented with 8 μl of DSB assay master mix to give a final reaction volume of 10 μl. In addition to beads from affinity enrichment, always one negative control with water, one positive control with 100 U T4 DNA ligase (NEB) and one reaction with 5 μg HEK 293T cell lysate was included. Positive and negative control were incubated for 30 min at 17°C, HEK 293T cell lysate and affinity enrichment reactions were incubated for 60 min at 17°C. After incubation at 17°C, all reactions were stopped by heating to 65°C for 15 min followed by addition of stopping solution (80% (v/v) formamide, 20 mM EDTA, 0.025% (w/v) bromophenol blue, and 0.025% (w/v) xylene cyanol) and heating to 95°C for 5 min. Samples were resolved by 8% denaturing urea-PAGE and analyzed by autoradiography. The assay was performed in biological triplicates. Dimer band intensities were evaluated using Image Lab V6.1 (BioRad) and calculated in ratio to the corresponding lysate control. GraphPad Prism V6 (GraphPad Inc.) was used for statistical analysis. A repeated measures one-way ANOVA with Tukey's multiple comparison test was performed with a confidence level of 0.05.

## Results and discussion

**Figure 1.**
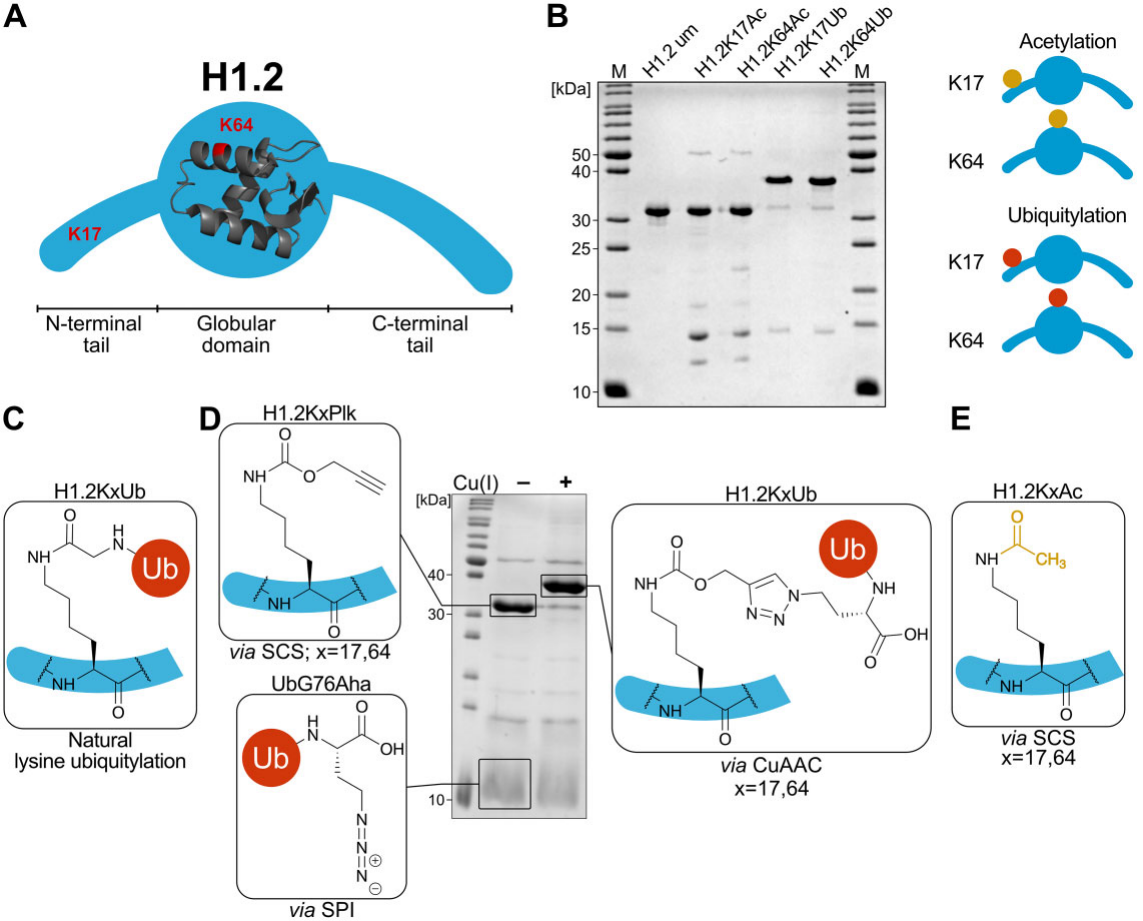
Site-specifically ubiquitylated and acetylated H1-variants generated for this study. (**A**) Schematic structure of linker histone H1.2, lysine residues at position 17 and 64 are indicated in red. The structure of the globular domain was obtained from PDB structure 8H0V (64). (**B**) Schematic overview and SDS-PAGE of all H1.2 variants generated for this study. um: unmodified; Ac: acetylated; Ub: ubiquitylated. (**C**) Chemical structure of the naturally occurring isopeptide bond in lysine ubiquitylation. (**D**) Preparation of site-specifically ubiquitylated H1.2 by Cu(I)-catalyzed azide-alkyne cycloaddition (CuAAC). An alkyne moiety is introduced into H1.2 by incorporation of the unnatural amino acid Plk *via* stop codon suppression (SCS) and an azide moiety is introduced into ubiquitin *via* selective pressure incorporation (SPI). H1.2 and ubiquitin are conjugated by CuAAC resulting in the stereoselective formation of a bioorthogonal 1,4-substituted triazole linkage. (**E**) Site-specific acetylation of H1.2 by introduction of N_ϵ_-acetyl lysine *via* SCS.

The human linker histone variant H1.2 was chosen for our studies because of its ubiquitous expression. H1.2 exhibits a tripartite structure, which is shared with other linker histone variants, composed of a central globular domain flanked by N- and C-terminal tails (Figure 1A). Lysine 17 in the N-terminal tail and lysine 64 in the globular domain of H1.2 were previously found to be both ubiquitylated and acetylated in cells (5,6,14,15,39,40). We therefore generated versions of H1 that were either acetylated or ubiquitylated on lysine 17 or 64, resulting in 4 differently modified variants in addition to unmodified H1.2 (Figure 1B, Supplementary Figure S1) (14,15). Ubiquitylation was achieved by conjugation of azide-modified ubiquitin with alkyne-containing H1.2 by copper-catalyzed azide-alkyne cycloaddition (CuAAC, click reaction) with near quantitative conversion (Figure 1D). The site-specific introduction of an alkyne moiety in H1.2 was achieved by incorporation of the unnatural amino acid Plk, a propargyl-derivative of lysine, during protein expression via amber stop codon suppression (SCS), producing H1.2KxPlk (x = 17, 64) (15,28,29). Ubiquitin containing a C-terminal azide moiety was prepared by introduction of azidohomoalanine (Aha) through selective pressure incorporation (SPI) to give UbG76Aha (29). The 1,4-substituted triazole linkage resulting from CuAAC between H1.2KxPlk and UbG76Aha (Figure 1D) is a bioorthogonal alternative to the natural isopeptide bond between the C-terminal carboxy group of ubiquitin and the ϵ-amino group of a target protein's lysine while retaining similar electronic properties (Figure 1C) (41). Acetylated H1.2 was prepared by the site-specific incorporation of N_ϵ_-acetyl-lysine through SCS at position 17 or 64 of H1.2 successfully yielding H1.2KxAc (*x* = 17, 64; Figure 1E) (14). All H1.2 variants were purified using a C-terminal His_6_ tag, ensuring purification of full-length expression products.

**Figure 2.**
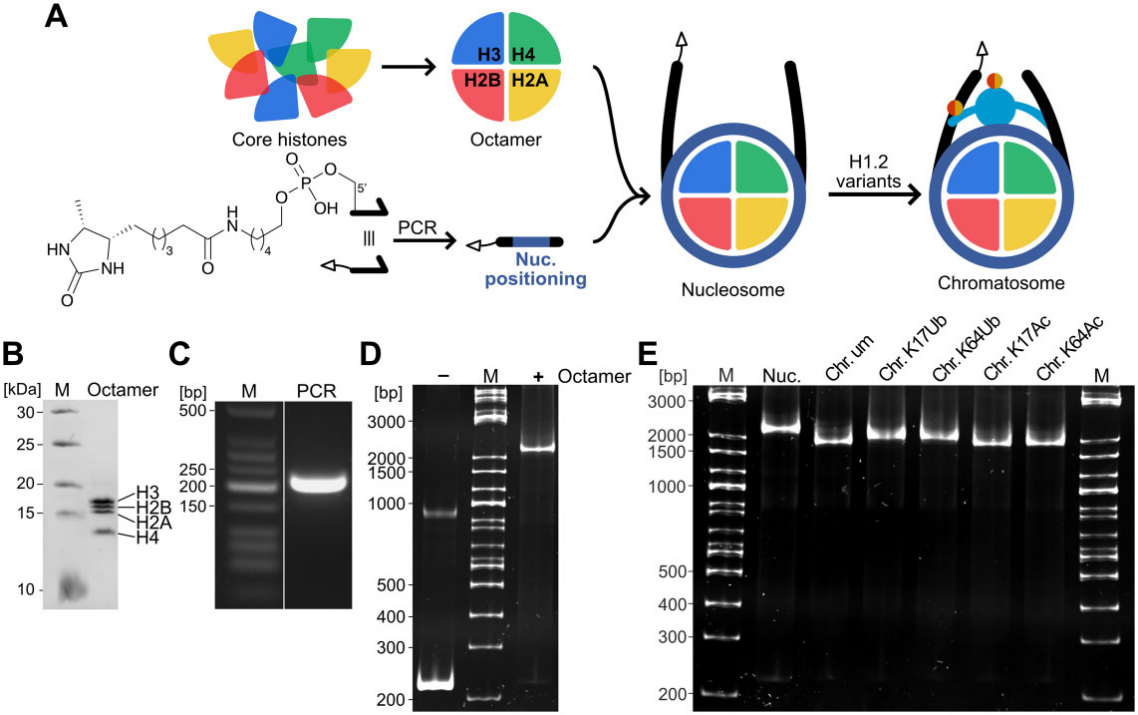
Generation of chromatosome variants with site-specifically ubiquitylated and acetylated linker histone H1.2. (**A**) Schematic overview of affinity-tagged chromatosome assembly using site-specifically modified H1.2 variants from building blocks. Core histones were expressed in *E. coli* and assembled into the histone octamer. A desthiobiotin affinity tag was introduced into the nucleosomal DNA by 5′-primer modification used in PCR amplification of nucleosomal DNA. Nucleosomes were subsequently assembled by mixing of core histone octamers and desthiobiotin-labeled DNA with a nucleosome positioning sequence. Chromatosomes were obtained by addition of site-specifically modified H1.2 variants. (**B**) SDS-PAGE gel of assembled core histone octamer shows the presence of all core histones in equal parts. (**C**) Agarose gel of PCR reaction after amplification of nucleosomal DNA shows specific production of the desired product (207 bp) in high quantities. (**D**) Native PAGE gel of assembled nucleosomes shows quantitative formation of nucleosomes upon addition of core histone octamer to nucleosomal DNA. (**E**) Native PAGE gel of intact chromatosome variants demonstrates quantitative conversion of nucleosome into chromatosome H1.2 variants.

To investigate the influence of a specific PTMs of H1.2 on PPIs in the context of the chromatosome, the assembled chromatosomes used as baits needed to be as homogeneous as possible. Thus, human core histones H2A, H2B, H3 and H4 were expressed and purified from *E. coli* (Figure 2A, Supplementary Figure S2A, S2D) and used to assemble the octamer (Figure 2B, Supplementary Figure S2B, C) (21,22). The nucleosomal DNA was generated by PCR and was equipped with a desthiobiotin (DTB) affinity tag introduced via a 5′-modified primer during PCR amplification (Figure 2A, 2C). Nucleosomes were assembled by combining the DNA with the core histone octamer in high salt buffer, followed by stepwise dialysis to low salt buffer (Figure 2D, Supplementary Figure S3A) (21). Nucleosome assembly was monitored with native PAGE, indicating successful and quantitative assembly. Finally, chromatosomes were quantitatively assembled by adding H1.2 variants to the previously assembled nucleosomes (Figure 2E, Supplementary Figure S3B). Interestingly, we observed that the chromatosome migrated faster than the nucleosome in native PAGE, although its larger size would suggest the opposite (Figure 2E) (14). This is likely due to the nucleosomal DNA that contains an additional 30 bp of linker DNA on either side of the central 147 bp that span around the octamer. This results in the formation of a very compact chromatosome structure and thus faster migration in native PAGE and the same migration behavior has been observed previously when nucleosomal DNA of comparable length was used (42). When ubiquitylated H1.2 was added to nucleosomes, this migration behavior was partially reversed, likely due to the additional protein mass of ubiquitin (Figure 2E).

**Figure 3.**
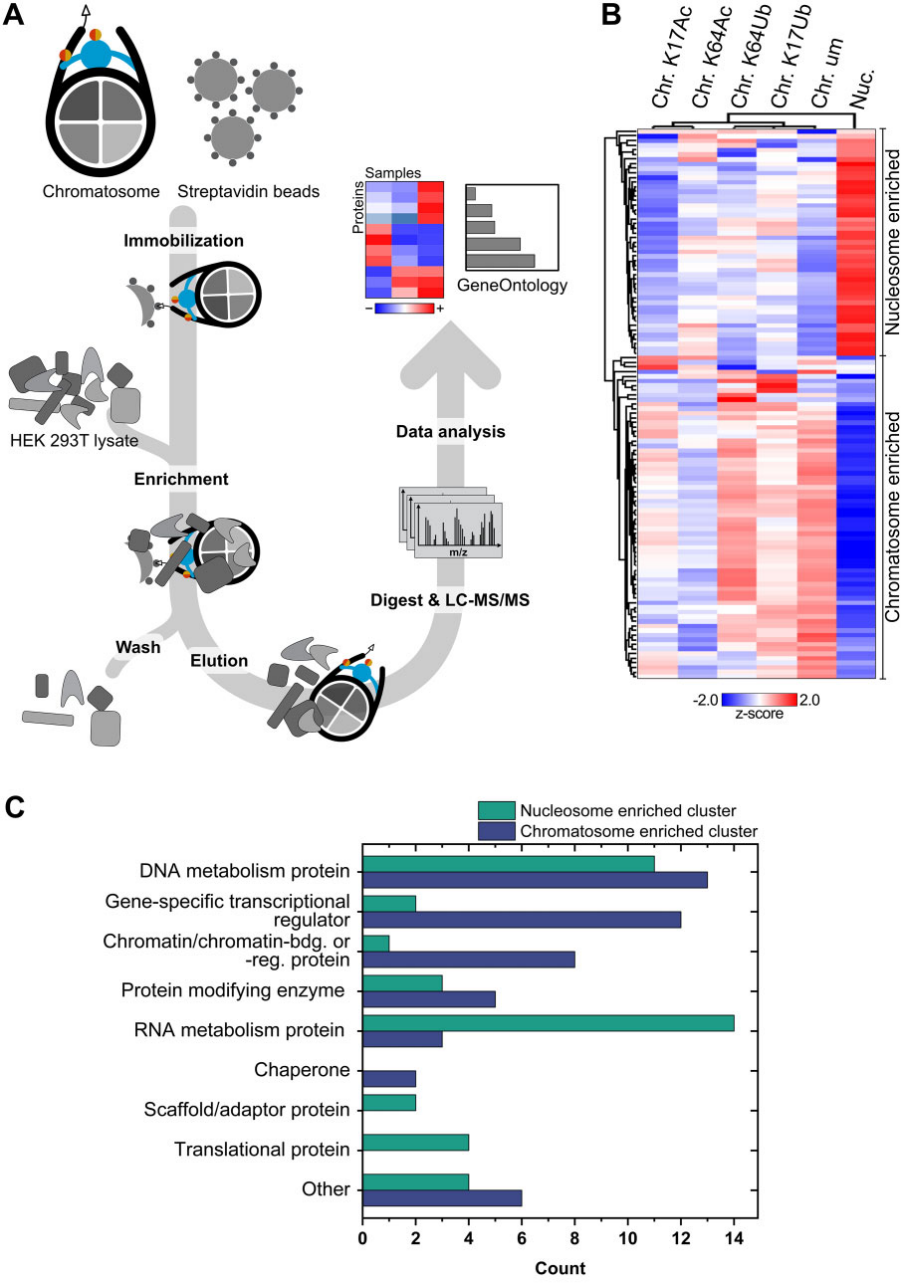
Interactome of H1-variant specific chromatosomes. (**A**) Overview of AE-MS workflow for H1.2 variant-specific chromatosome interactomes. Chromatosomes containing unmodified H1.2 or H1.2 variants are immobilized on streptavidin beads via a desthiobiotin tag on the nucleosomal DNA. Interacting proteins are enriched from HEK 293T cell lysates followed by washing steps to remove unspecific binders. After gentle elution of intact chromatosomes and enriched interactors with biotin, proteins are subjected to a tryptic digest followed by identification by LC-MS/MS and label-free quantification using DIA. Data analysis and statistical validation reveals PTM-specific interactions. (**B**) Heatmap of significantly enriched interactors for nucleosomes and chromatosome H1-variants. Shown are the mean z-score normalized values for each sample (ANOVA *S*_0_= 0.1, FDR = 0.05; *t*-test *S*_0_= 0.1 FDR = 0.05). (**C**) Classification of enriched proteins by GO-terms using PANTHER for the nucleosome (green) and chromatosome (blue) cluster.

With the successfully assembled chromatosomes in hand, we then optimized our affinity enrichment procedure (Figure 3A). Chromatosomes were first immobilized on streptavidin-coupled beads to allow removal of any unbound material and residuals from prior assembly procedures by washing. To verify that chromatosome stability was maintained during the procedure, chromatosomes immobilized on beads were incubated with HEK 293T lysate simulating an affinity enrichment experiment. Samples taken at different time points showed an unchanged migration behavior of the chromatosomes in native PAGE after elution with biotin, indicating suitable stability even after incubation in cell lysate for 20 hrs and demonstrating that quantitative immobilization and elution of intact chromatosomes from beads is possible (Supplementary Figure S3C).

The affinity enrichment of interacting proteins was carried out as shown in Figure 3A, using biological triplicates for all our seven samples: chromatosomes containing unmodified H1.2 or H1.2K17Ub, H1.2K64Ub, H1.2K17Ac or H1.2K64Ac variants, as well as nucleosomes and empty beads as control. Enriched proteins were identified and quantified using a label-free data independent acquisition (DIA) MS workflow with variable window size. This procedure was optimized for our approach (Supplementary Table S3) allowing us to reliably quantify approximately 1500 proteins across all bait samples (Supplementary Figure S4A). Evaluation of the data with principal component analysis (PCA) indicated large differences between samples and the empty bead control, in comparison to minimal variability in-between baits (Supplementary Figure S4B). Since these differences prohibited direct evaluation of significant differences between baits, all proteins that were significantly enriched for any of the bait samples were first determined through ANOVA, resulting in 728 proteins (*S*_0_ = 0.1, FDR = 0.05; Supplementary Figure S4C). Significant enrichment for a specific chromatosome variant was in a next step then determined by pairwise comparison to the nucleosome sample, resulting in 119 significantly enriched proteins (*S*_0_ = 0.1, FDR = 0.05, *t*-test; Supplementary Figure S5). These could then be further divided into two main groups: a nucleosome-enriched cluster of 49 proteins and a chromatosome-enriched cluster of 70 proteins (Figure 3B).

Overall, the associated protein classes, as obtained by PANTHER (35) were as expected for chromatin interacting proteins, thus validating our tailored affinity enrichment approach (Figure 3C). We find that within the nucleosome-enriched cluster, a high number of proteins were associated with RNA metabolism, which is due to the presence of 8 subunits of DNA-dependent RNA polymerases I to III and 5 subunits of the exosome complex in this cluster. Enrichment of RNA polymerase subunits only in the absence of H1.2 is in agreement with previous findings of inhibitory effects of H1.2 on transcription (43). The presence of the exosome complex is consistent with the enrichment of RNA polymerases, as the complex is a known RNA processing factor.

Besides inhibitory effects of H1.2 on transcription also an activating role has been observed (7) which is reflected in our data by the chromatosome-specific enrichment of proteins classified as gene-specific transcriptional regulators, albeit with no preference for any of the PTMs on H1.2.

Identification of various regulatory transcription factors enriched for chromatosomes indicates a role of H1.2 in the regulation of transcription initiation, while the actual transcription process performed by DNA-dependent RNA polymerases preferentially occurs in absence of H1.2. Not surprisingly, half of the chromatosome-enriched transcriptional regulators were annotated to contain at least one zinc finger domain. Based on this observation, all proteins were further evaluated and 16 out of 70 proteins within the chromatosome-enriched cluster contained a zinc finger domain, compared to only 4 out of 49 for the nucleosome-enriched cluster. Since all affinity enrichment samples contained the same DNA sequence, this finding hints at an epigenetic role of zinc finger proteins that does not solely depend on their DNA binding capabilities and may encompass H1.2 binding. Apart from transcription-related proteins, DNA-replication associated proteins such as 3 subunits of replicative MCM helicase or topoisomerases II alpha and beta were enriched for chromatosomes, also supporting the view of H1.2 as regulator beyond chromatin compaction.

Most proteins within the chromatosome-enriched cluster were preferentially binding to unmodified H1.2, but over 40% of the proteins were enriched either additionally or solely for chromatosomes containing modified H1.2 (Supplementary Figure S4D). No unique enrichment was observed for chromatosomes containing acetylated H1.2, which could be explained by the need for the presence of additional epigenetic marks for cellular effects of H1.2 acetylation. Ubiquitylation of H1.2 on the other hand had a more distinct impact on the chromatosome interactome. In total, besides ubiquitin we have identified six ubiquitylation-specific interactors - SERF2, VPS25, DCUND1D1, BARD1, MTA1 and USP16 (Supplementary Figure S4D).

**Figure 4.**
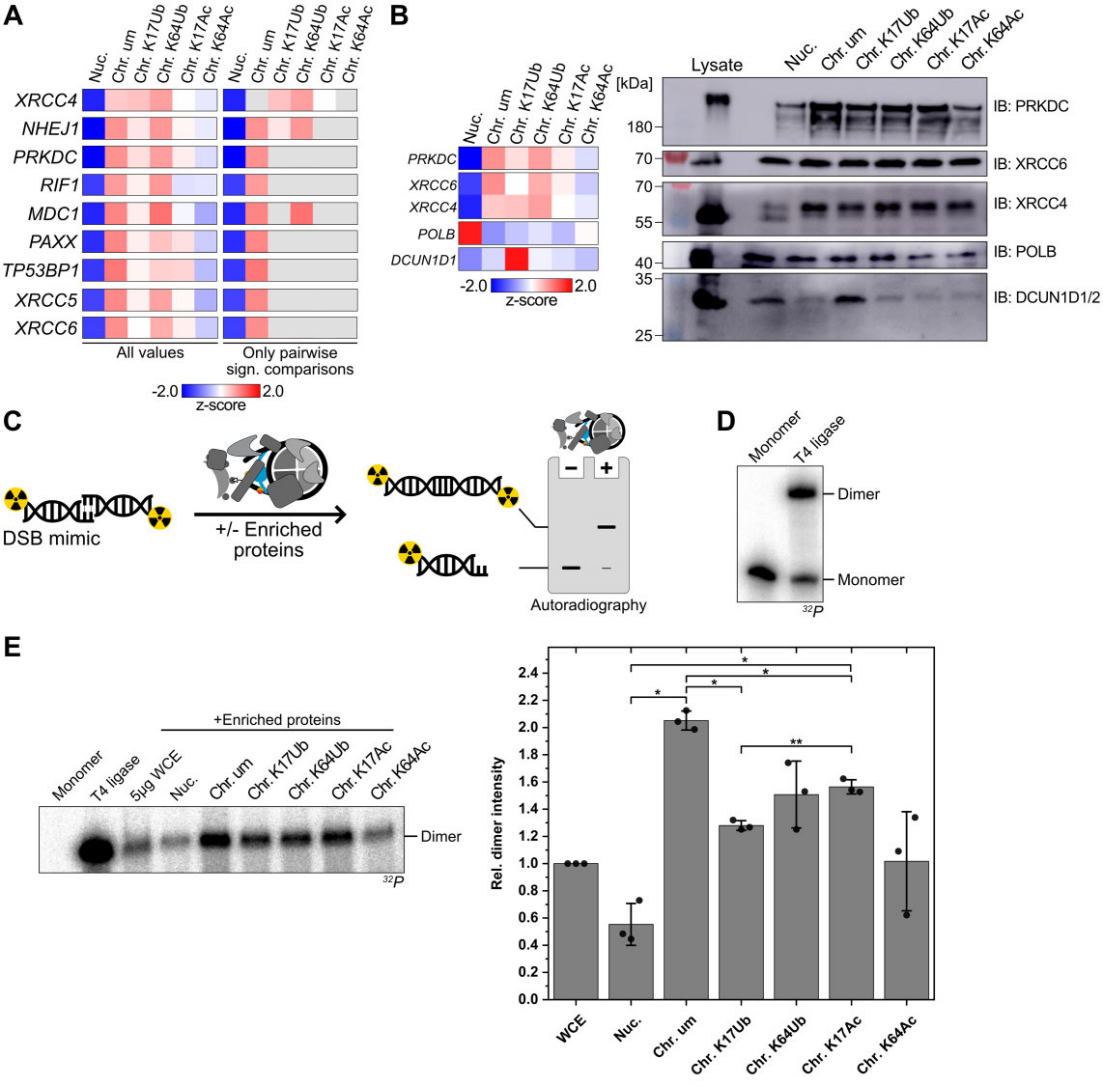
Functional validation of chromatosome-specific interactors involved in DSB. (**A**) Heatmap of proteins associated with the NHEJ pathway of DSB repair. Shown are the mean *z*-score normalized values for each sample (left panel). In the right panel, only those values are shown where the pairwise comparison between the nucleosome and the respective chromatosome variant showed significant enrichment (*t*-test, S0 = 0.1, FDR = 0.05). (**B**) Confirmation of protein enrichment by immunoblotting. Enrichment was performed as in Figure 3, but elution was performed with SDS loading dye followed by SDS-PAGE and western blotting. Indicated are the primary antibodies used for protein visualization. Left: Excerpt of heatmap as shown in Figure 3B to highlight relative enrichment of proteins involved in DSB in our AE-MS data. (**C**) Schematic representation of DNA DSB assay with radioactively labeled DNA. 5′-^32^P labeled DNA duplex with a self-complementary 3′-overhang serves as DSB mimic. Enrichment of bait interacting proteins is performed as depicted in Figure 3, then beads with enriched proteins are incubated with the radioactively labeled DSB mimic. In the presence of ligation-proficient proteins, two DNA duplexes are ligated, resulting in a marked shift visible via denaturing PAGE and autoradiography. (**D**) Autoradiography assay: Monomer: negative control, incubation with water, T4 ligase: positive control, addition of 100 U T4 DNA ligase. Upon addition of the ligase, clear formation of the ligation product (upper band) is visible. (**E**) Left: Autoradiography of denat. PAGE of the DSB assay carried out in the presence of beads with immobilized bait and enriched proteins. Monomer & T4 ligase are controls as described in (D), 5 μg WCE: incubation with 5 μg HEK 239T whole cell extract (WCE). Right: result of quantification and statistical analysis of triplicates of the DSB assay. The dimer band intensity was calculated relative to the WCE sample lane to compensate for differences in lysate activity. Shown is the mean and standard deviation as well as individual values for all biological replicates (*n* = 3). Significant differences were determined by one-way repeated measures ANOVA and Tukey's multiple comparison test, *P< 0.05, **P< 0.01.

To the best of our knowledge, up to now an interaction with ubiquitylated H1 has not been established for any of these proteins. For three of these proteins (SERF2, VPS25 and DCUND1D1), also a connection to chromatin or unmodified H1 has not been reported so far. To corroborate our findings, we validated DCUND1D1 and confirmed the specific enrichment for H1.2K17Ub chromatosome variants by western blot analysis (Figure 4B). The BRCA1-associated BARD1 protein, the transcriptional coregulator MTA1, and the ubiquitin-specific protease USP16 were all enriched for H1.2K64Ub chromatosomes in our study. For MTA1, it has been shown that it plays a role in the regulation of chromatin structure, but so far a physical interaction with H1 has not been reported (44). Furthermore, there exists a relatively large body of work that shows that BARD1 together with BRCA1 recognizes H2AK15-ubiquitylated nucleosomes in the event of DNA damage (45). Our data may suggest that the BARD1/BRCA1 complex recognizes ubiquitylated chromatin in general. Future studies will therefore need to explore the role of H1 ubiquitylation in DNA damage (46) and investigate if BARD1 is able to directly detect ubiquitylated H1 or if a more indirect mechanism is taking place. USP16 has been linked to deubiquitylation of H2A (47), but so far no connection to linker histone H1 has been identified. This could indicate that H1.2K64Ub may function as another target of USP16 or that H1.2K64Ub serves as docking site for USP16 for subsequent H2A deubiquitylation.

Several enriched proteins have known roles in DNA repair and interestingly, the preferential enrichment for either nucleosomes or chromatosomes was dependent on the specific DNA repair pathway they are involved in. Among nucleosome-enriched proteins were the exonuclease *EXO1*, which is involved in multiple repair processes (48), and DNA polymerase β, which is involved in base excision repair (49). Furthermore, proteins of the nucleotide excision repair (NER) pathway were also preferentially interacting with nucleosomes and here especially proteins, that carry out functions in more advanced steps of the repair process. Among these, the helicase XPB, a part of the TFIIH complex, as well as the nucleases XPF and XPG were identified, supporting a model where the actual repair of DNA damage by NER would occur in the absence of H1.2 and thus in a more open chromatin environment (50). Yet, RAD23B, part of a DNA damage sensing complex during NER (50) was present in the chromatosome-enriched cluster, suggesting a role for H1.2 in this process of damage recognition.

Finally, proteins associated with the repair of double strand breaks (DSBs) *via* both known major pathways, homologous recombination (HR) and non-homologous end-joining (NHEJ), were enriched for chromatosomes. A potential explanation for the preferred binding of DSB repair proteins to chromatosomes is the blunt-ended dsDNA, which is part our affinity enrichment baits and resembles a DSB. Specific enrichment of repair proteins only in presence of H1.2 variants however indicates an important role of H1.2 in the recruitment of repair proteins to DSB sites. In the case of HR, the two end-resection promoting proteins PSIP1 (51) and HDGFR2 (52) as well as POGZ, an interaction partner of HDGFR2 (52) were enriched as were all three heterochromatin binding proteins HP1 α, β and γ. Specific enrichment of HP1 proteins in the presence of H1.2 suggests that their recognition of heterochromatin may have an H1.2-based component in addition to their known binding to heterochromatin marks on core histones (53).

A Fisher exact test revealed that almost all proteins annotated with a role in NHEJ were enriched specifically for chromatosomes and here in particular by chromatosomes containing either unmodified H1.2, any of the ubiquitylated variants, or K17 acetylated H1.2 (Figure 4A, Supplementary Figure S5). Chromatosomes containing K64 acetylated H1.2 in contrary showed less enrichment for NHEJ proteins. Ubiquitylation of linker histones with low site-specificity has been found to occur in response to DNA damage induced by UV irradiation previously (46). Since acetylation of lysine 64 prohibits its ubiquitylation, impeded binding of the DSB repair machinery might indicate that of the investigated sites, lysine 64 is the one with the greater functional impact.

Among the enriched DSB repair proteins were *XRCC5* and *XRCC6* (Ku70, Ku80), which are all involved in DSB recognition and the regulating protein *TP53BP1* (54) and references therein), but also proteins involved in later steps of NHEJ like the catalytic subunit of the DNA-dependent protein kinase, known to exert a regulatory function and *XRCC4* which is needed for the final ligation step in complex with DNA ligase IV (54) and references therein).

In order to further support the results obtained by our AE-MS experiments, we validated specific PPIs by immunoblotting using primary antibodies against several enriched proteins (Figure 4B and Supplementary Figure S6). For *DCUN1D1*, our data confirmed the specific enrichment for H1.2K17Ub chromatosomes, and for *XRCC4* and *XRCC6* we could show preferential enrichment for chromatosomes in general (Figure 4B and Supplementary Figure S6). The immunoblotting results thus overall support our mass spectrometry-based results.

Based on our observation that nearly all the identified proteins from our AE-MS experiments with a known role in the NHEJ repair pathway showed a preferential enrichment for chromatosomes, we wanted to functionally validate this observation. We therefore set-up an assay that allowed us to study the ligation of two double-stranded oligonucleotides *in vitro* in a semi-quantitative manner and used the ligation reaction as a proxy for a DSB (Figure 4C). Within this assay the enrichment of interacting proteins was performed as in our AE-MS experiments, but instead of eluting the proteins, these were then incubated with a radioactively labeled DNA substrate which had previously been shown to be preferentially repaired by proteins of the NHEJ pathway (37,38). Due to a self-complementary overhang, two of these DNA substrate molecules are ligated in the presence of DNA repair enzymes (Figure 4C), resulting in a marked shift in PAGE analysis (Figure 4D). The repair efficiency was then assessed by autoradiographic quantification of the band intensity of the dimeric ligation product and calculation of the relative dimer band intensity in comparison to a control, in which the DNA substrate molecules were incubated with HEK 293T lysate. As negative control, the repair assay was performed with immobilized nucleosomes or chromatosomes that were not preincubated with cell lysate, which did not result in any formation of the dimeric ligation product (Supplementary Figure S7A).

Applying our assay in triplicates (Supplementary Figure S7B), we found statistically significant differences between chromatosome and nucleosome samples in their efficiency to promote DNA ligation. All chromatosome variants showed a significantly higher propensity to promote the ligation of double-stranded oligonucleotides than nucleosomes, in line with the differential enrichment of NHEJ DSB repair proteins seen in our AE-MS data (Figure 4E, Supplementary Figure S7C). When comparing the differently modified chromatosome variants relative to each other, acetylation of lysine 64 of H1.2 had the lowest effect on promoting DNA ligation. This effect was as well reflected in our AE-MS data, where chromatosomes containing H1.2K64Ac showed the least enrichment for DSB repair proteins. While linker histones have up to now been associated with an inhibitory role in the final ligation step of DSB repair by NHEJ (55) our observations suggest that H1.2 may be rather required in the vicinity of DSBs to bind the proteins needed for DNA repair.

When comparing the results obtained from chromatosome affinity enrichments with previous studies that have investigated the interactome of H1.2 on its own - site-specifically modified at the same positions as in this work (14,15)—a relatively minor overlap between identified interactors could be observed. Overall, 5 of the previously identified specific interactors of free H1 remained significantly enriched for at least one of the chromatosome variants when we applied our stringent filtering (Supplementary Figure S8). This corresponds to 17% of the proteins that we have identified as interactors of posttranslationally modified (i.e. ubiquitylated or acetylated) H1.2 in this work.

It could be that proteins that only interact with free modified or unmodified H1.2 bind to regions of H1.2 that otherwise interact with DNA in chromatosomes. However, interactions between linker histones and DNA have been shown to occur between the globular domain of H1 as well as both N- and C-terminal H1 tails (43). As the interaction appears to be mainly mediated by contacts between positively charged amino acid residues within H1 and the negatively charged phosphate backbone of DNA, no distinct DNA binding domain seems to exist for linker histones. Another possibility to explain the different binding behavior would be that H1 undergoes a conformational change upon chromatosome formation. Along this line, it has been reported that binding of unmodified H1 to nucleosomes affects the flexibility of the linker DNA (56) and this altered accessibility of nucleosomal DNA might also result in changes in the interaction behavior. Nonetheless, based on our robust and highly reproducible AE-MS data we interpret these fairly separate sets of H1-PTM-specific interaction partners to strengthen the view of H1 as a protein with distinct roles depending on its occurrence in the cell (free or chromatin-bound), an interpretation that is supported by previous observations (57,58).

Among the proteins that were found to be enriched for both free H1.2 and chromatin-associated linker histone were the histone chaperone NAP1L4, which showed preferential binding to H1.2K17Ac chromatosomes, and nucleoplasmin 3 which showed preferential binding to chromatosomes containing either of the acetylated H1.2-variants. Interestingly, both of these proteins had previously been identified to be enriched for unmodified or ubiquitylated H1.2. Conversely, for the kinase NOL9 which exhibited previously preferential enrichment for unmodified or acetylated H1.2, we now see stronger binding to unmodified or ubiquitylated chromatosome variants. Also, for the UV excision repair protein RAD23B, which had previously been identified to be enriched for ubiquitylated H1.2, we now see no preference for any of our chromatosome variants. (57,58).

A search for annotated domains in uniprot for those proteins that were specifically enriched for chromatin-bound H1 showed that multiple of these proteins contain bromo- or chromodomains that are known to bind to acetylated and methylated lysine residues in core histones (59,60) (Supplementary Table S4). In contrast, for the proteins that were previously found to be enriched for free H1.2 (14,15), only few were shown to interact with chromatin components or to possess chromatin-interacting chromo- or bromodomains. It is therefore likely that many of the chromatosome-specific binders recognize chromatosome elements besides the modified linker histones. As we have employed intact chromatosomes as baits in our study, we cannot exclude that the interaction has been mediated by nucleosomal DNA or via another interactor rather than by direct H1.2 contacts. Yet, our study demonstrates that the presence of (modified) H1 is necessary for certain interactions to occur, as we observed specific enrichment for these proteins when using chromatosomes, and not nucleosomes, as baits.

## Conclusions

Here, we report on a tailored AE-MS platform for the study of PPIs of chromatosomes with site-specifically modified linker histone variants on a proteome-wide scale. Our AE-MS experiments provide novel insights into the cellular interaction partners of these chromatosome variants and reveal specific interactors of chromatin bound linker histone. The identification and validation of both modification- and site-specific PPIs in our study further supports the emerging picture of modular cellular interactomes that are driven not only by a given modification but also depend on the *specific location* of the respective PTM (14,15,61,62). Interestingly, proteins necessary for NHEJ of DNA double strand breaks preferentially bound to the various chromatosomes variants but not mere nucleosomes. This observation was independently validated by immunoblot analysis and functionally confirmed by an *in vitro* ligation assay. This unexpected finding is a starting point for further research into the role of H1.2 in response to DNA damage and particular in DSB repair.

Previous interactions studies using intact proteins as baits have either focused on modifications of core-histones (13) or employed only nucleosomes as baits (10,12,63). Thus, to the best of our knowledge our study is the first that uses intact chromatosome variants as baits for AE-MS to elucidate H1-PTM-specific interactions, making a direct comparison with other studies difficult. A direct comparison of the present study with our previous studies (14,15), which employed free H1.2 that was site-specifically ubiquitylated at the same positions as the H1.2 variants used here, suggests different roles of ubiquitylated H1 when it is free (i.e. not bound to nucleosomes) in the cell or functions as part of chromatin. It also underscores the importance of investigating chromatin and chromatin PTMs with probes that closely approximate the complexity of native chromatin.

More generally, our modular chromatosome-baits assembled from building blocks and optimized workflow constitutes not only an important tool for future MS-based proteomics investigations of histone epigenetic modifications. Taken together our study supports the role of chromatin-bound H1.2 as a regulatory protein with distinct functions beyond DNA compaction and functions as an important resource for future investigations of histone epigenetic modifications.

## Supplementary Material

gkad1113_Supplemental_Files

## Data Availability

The mass spectrometry proteomics data have been deposited to the ProteomeXchange Consortium via the PRIDE (36) partner repository with the dataset identifier PXD040236.
