# Supplementary material for: Interactome of intact chromatosome variants with site-specifically ubiquitylated and acetylated linker histone H1.2
Source: Nucleic Acids Res. 2023 Nov 22;52(1):101–13. doi: 10.1093/nar/gkad1113 (PMC10783519; doi:10.1093/nar/gkad1113)
Supplement: gkad1113_Supplemental_Files [file gkad1113_supplemental_files.zip › Supplementary Information.pdf]

Supplementary Information

Interactome of intact chromatosome variants with site-specifically ubiquitylated and acetylated linker histone H1.2

Authors:

Philip Saumer[1,2], Martin Scheffner[2,3], Andreas Marx[1,2*] and Florian Stengel[2,3]

Affiliations:

[1]Department of Chemistry, University of Konstanz; Universitätsstraße 10, 78464 Konstanz, Germany.

[2]Konstanz Research School Chemical Biology; University of Konstanz, Universitätsstraße 10, 78464 Konstanz, Germany.

[3]Department of Biology, University of Konstanz; Universitätsstraße 10, 78464 Konstanz, Germany.

*Corresponding authors emails: andreas.marx@uni-konstanz.de & florian.stengel@uni-konstanz.de

Table of Contents

Supplementary Tables 3
Supplementary Figures 8
Supplementary References 16

Supplementary Tables

Table S1: Oligonucleotides for the PCR of desthiobiotin (DTB)-labeled nucleosomal DNA and for the double strand break (DSB) repair assay.

Oligonucleotide	Sequence 5‘ – 3‘
Nuc. DNA forward	ATCTAATACTAGGACCCTATACGC
Nuc. DNA reverse	*DTB*-TTTTTTTTTTTATCATTAA TATGAATTCGGATCCACATG
79mer (DSB Assay)	GTACGTAACTATGCTAATGATGGGTCTCGATTAGTCAGCAACCATAGT CTCAGTCCTAATTCCACTCATCCCGT CTCTA
75mer (DSB Assay)	TAGAGACGGGATGAGTGGAATTAGGACTGAGACTATGGTTGCTGACTA ATCGAGACCCATCATTAGCTAAGTTAC

Table S2: Amino acid sequences of the core histones used within this work. H2B and H4 are shown after TEV cleavage.

**H2A** (H2A 1-B/E)	SGRGKQGGKARAKAKTRSSRAGLQFPVGRVHRLLRKGNYSERVGAGAPVYLAAVL EYLTAEILELAGNAARDNKKTRIIPRHLQLAIRNDEELNKLLGRVTIAQGGVLPNIQA VLLPKKTESHHKAKGK
**H2B** (H2B 1-J)	GPEPAKSAPAPKKGSKKAVTKAQKKDGKKRKRSRKESYSIYVYKVLKQVHPDTGIS SKAMGIMNSFVNDIFERIAGEASRLAHYNKRSTITSREIQTAVRLLLPGELAKHAVSE GTKAVTKYTSAK
**H3** (H3.1)	ARTKQTARKSTGGKAPRKQLATKAARKSAPATGGVKKPHRYRPGTVALREIRRYQK STELLIRKLPFQRLVREIAQDFKTDLRFQSSAVMALQEACEAYLVGLFEDTNLCAIHA KRVTIMPKDIQLARRIRGERA
**H4**	SGRGKGGKGLGKGGAKRHRKVLRDNIQGITKPAIRRLARRGGVKRISGLIYEETRGV LKVFLENVIRDAVTYTEHAKRKTVTAMDVVYALKRQGRTLYGFGG

Table S3: DIA windows for measurement of affinity enrichment samples.

Window	Center [m/z]	Isolation width [m/z]
1	367.00000	34.0
2	397.00000	28.0
3	423.00000	26.0
4	447.50000	25.0
5	470.50000	23.0
6	492.50000	23.0
7	515.00000	24.0
8	537.00000	22.0
9	559.00000	24.0
10	581.50000	23.0
11	604.00000	24.0
12	627.50000	25.0
13	652.50000	27.0
14	679.00000	28.0
15	707.50000	31.0
16	738.50000	33.0
17	772.50000	37.0
18	812.00000	44.0
19	859.00000	52.0
20	920.00000	72.0
21	1013.00000	116.0
22	1360.00000	580.0

Table S4: Annotated chromatin-associated domains and interactions according to Uniprot for all chromatosome-enriched proteins identified in this work.

ADNP	
BAP18	Component of nucleosome remodeling factor complex (NURF)
BARD1	
BAZ1A	Contains Bromodomain; component of chromatin remodeling complex
BAZ1B	Same as BAZ1A
C5orf24	
CBX1	Contains Chromodomain; interaction with H3K9me
CBX3	Contains Chromodomain
CBX5	Contains Chromodomain
CDC37	
CDCA5	
CHAF1A	
CHAMP1	
CHD1	2 Chromodomains,
CHD9;CHD6;CHD8;CHD7	2 Chromodomains
CTCF	
DCUN1D1	
GTF3C1	
GTF3C3	
H1-2	
H1-3	
HDGFL2	Interaction with methylated lysins of H3
HDGFL3	
HMGA2	
LRWD1	WD repeat region; required for chromatin association
MCM2	Part of hexameric helicase complex; interaction with core histones H3.1 and H3.3
MCM3	Part of hexameric helicase complex; interaction with core histones H3.1 and H3.3
MCM5	Part of hexameric helicase complex; interaction with core histones H3.1 and H3.3
MDC1	Gamma H2Ax interactor; DNA damage recognition
MTA1	Component of nucleosome remodeling and deacetylase complex (NuRD)
NFIB;NFIC;NFIA;NFIX	
NHEJ1	
NLE1	
OARD1	
PAXX	
PBRM1	Contains several bromodomains; part of SWI/SNF-B chromatin remodeling complex.

PGAM1	
PHIP	Contains 2 bromodomains
PLS3	
POGZ	
PRKDC	
PSIP1	
PSMA3	
RIF1	
RPS27A	
RUVBL1	
SERF2	
SP1	
TFAP4	Transcription factor; DNA binding
TFCP2	Transcription factor; DNA binding
TMA7	
TOP2A	
TOP2B	
TP53BP1	
UBTF	
USP16	
VPS25	
XRCC4	
XRCC5	
XRCC6	
ZMYM3	May be part of histone deacetylase complex
ZMYND8	Contains bromodomain; H4K16Ac interaction
ZNF143	
ZNF428	
ZNF512	

Supplementary Figures

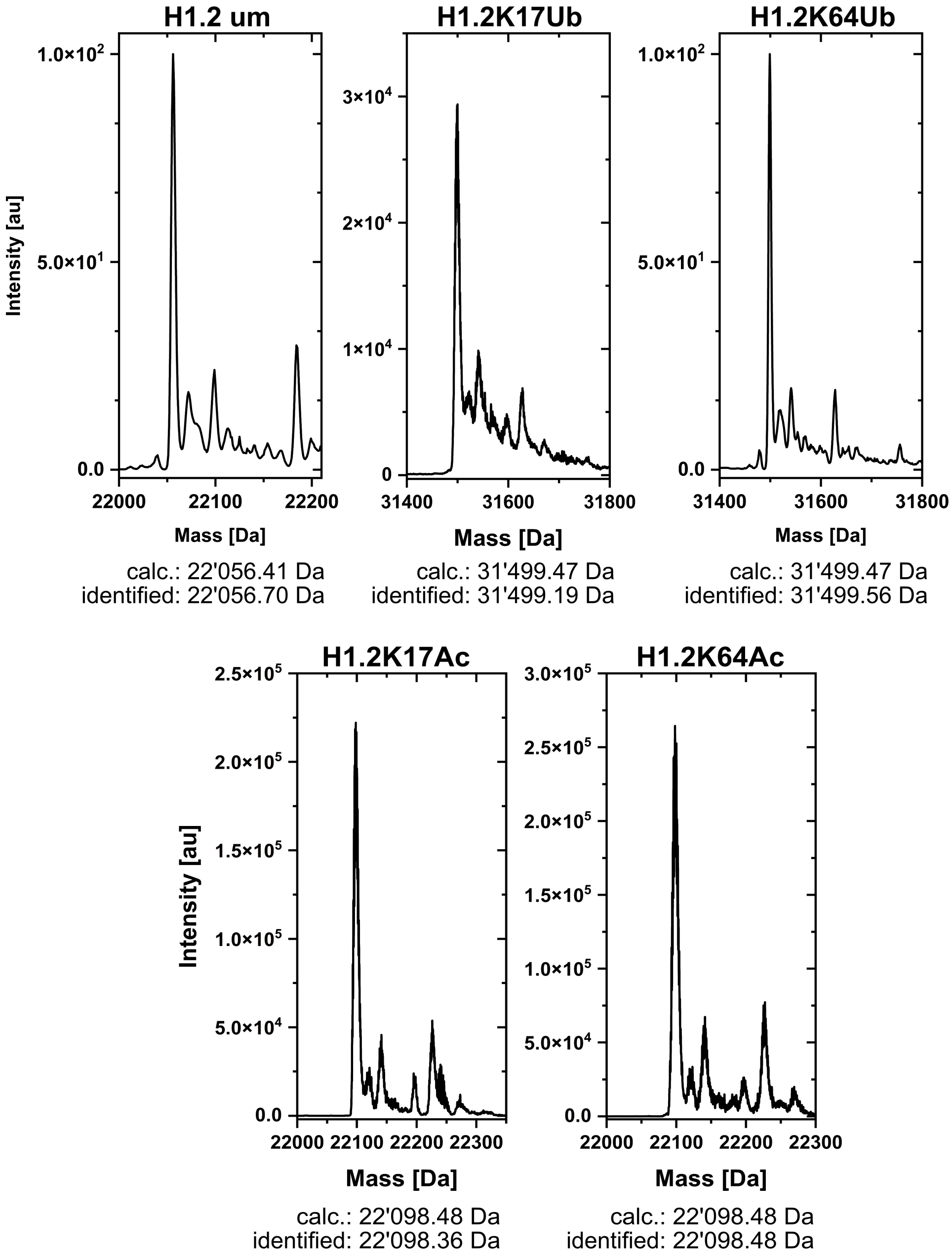

Figure S1: Deconvoluted ESI-MS spectra of purified H1.2 variants.

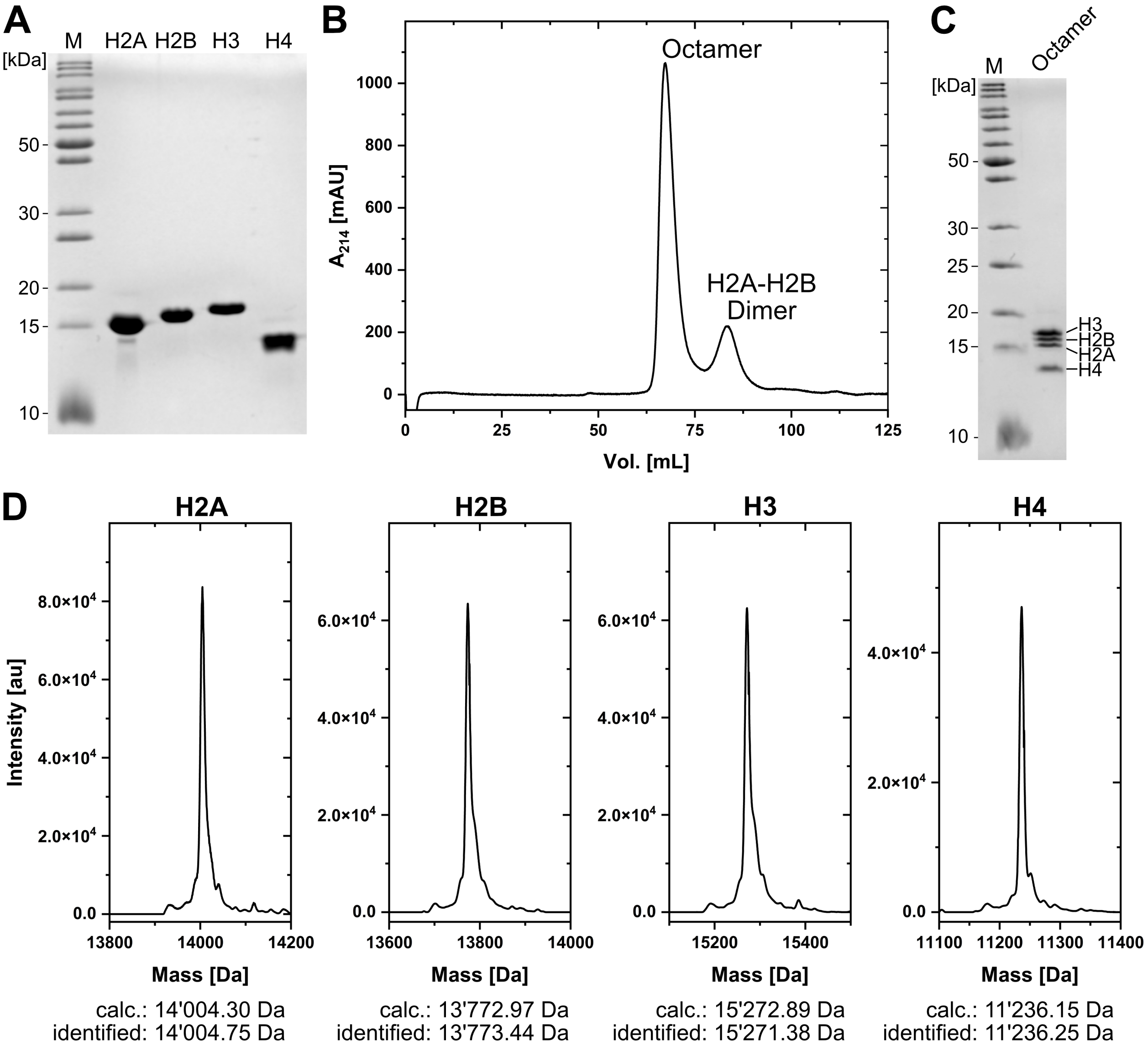

Figure S2: **Expression and purification of core histones and assembly of the core histone octamer.** A: SDS-PAGE of purified core histones. B: Chromatogram of core histone purification by size exclusion chromatography. C: SDS-PAGE of purified core histone octamer. D: Deconvoluted ESI-MS spectra of purified core histones.

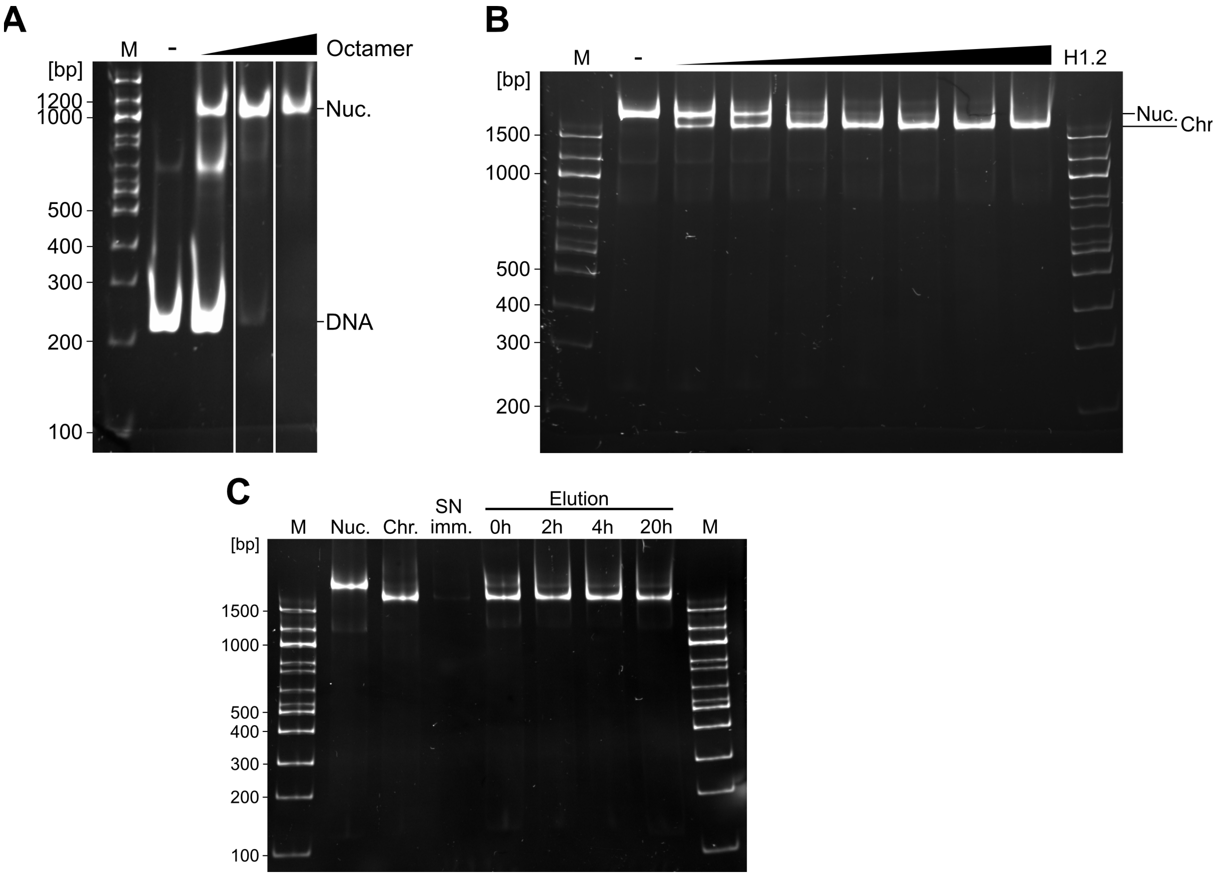

Figure S3: **Chromatosome assembly and evaluation**. A: Native PAGE gel of nucleosome (Nuc.) assembly. Titration of nucleosomal DNA (Nuc. DNA) and core histone octamer establish the optimal ratio for assembly. B: Native PAGE gel of chromatosome (Chr.) assembly. Titration of H1.2 to nucleosome to establish the optimal ratio for assembly. C: Native PAGE gel of chromatosome stability. Chromatosomes (Chr.) are quantitatively assembled from nucleosomes (Nuc.) and immobilized on streptavidin beads. Elution of intact chromatosomes with biotin is possible even after 20 hrs of incubation with HEK 293T cell lysate. SN Imm.: supernatant after immobilization, absence of any signal indicates quantitative immobilization on beads.

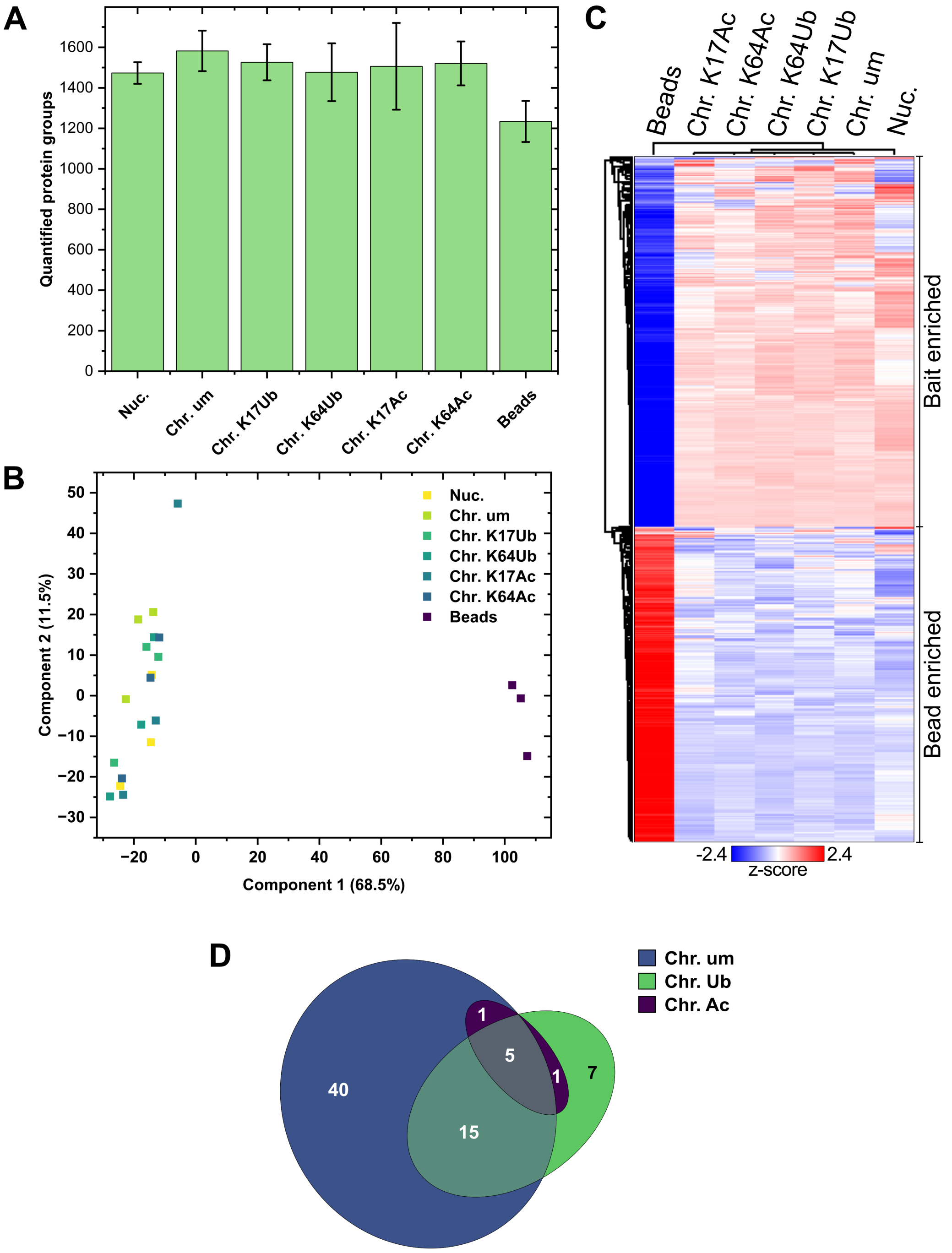

Figure S4: **Evaluation of affinity-enrichment MS workflow and data.** A: Number of quantified protein groups within each sample after filtering (≥ 4 valid values in at least one of the samples. Mean and standard deviations are shown (3 biological replicates, measured as technical duplicates). B: Principal component analysis (PCA) of all AE-MS samples. C: Heatmap of significantly enriched proteins for H1-variant chromatosomes (top) and control (bottom) (ANOVA; $S_0 = 0.1$, FDR = 0.05). Only the chromatosome-specific cluster (top) was used for further data evaluation. D: Venn diagram reveals overlap of significantly enriched interactors for unmodified H1 vs. ubiquitylated H1 and acetylated H1 – chromatosome variants (*t*-test; $S_0 = 0.1$, FDR = 0.05).

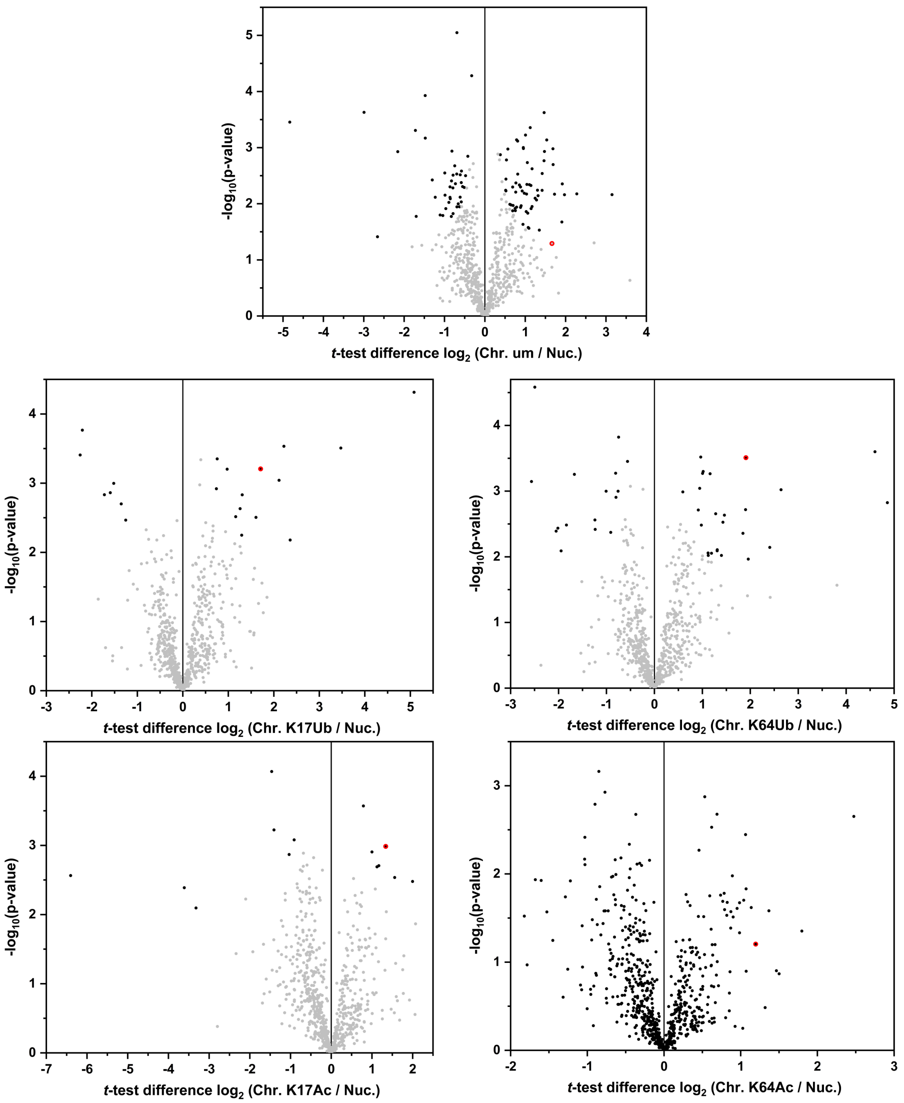

Figure S5: **Volcano plots of individual t-Tests.** Volcano plots of individual t-Tests performed to identify significantly enriched proteins for any of the chromatosome variants. Not-significant proteins are shown in grey, significant proteins in black. As example for NHEJ DSB repair proteins, XRCC4 is indicated in all t-Tests with a red circle.

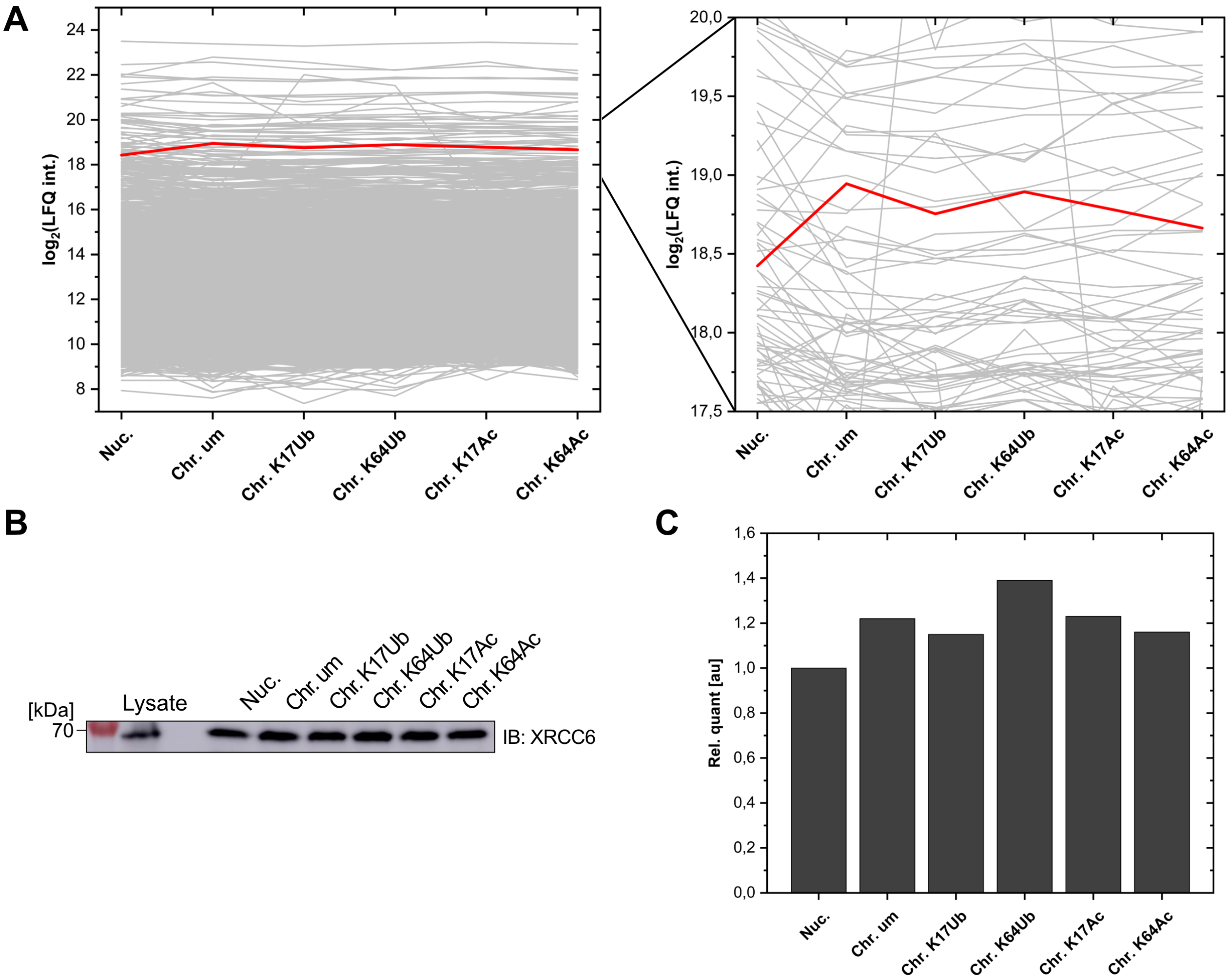

Figure S6: **Quantification of XRCC6 enrichment by MS and immunoblotting.** A: Log_2 transformed LFQ intensity of XRCC6 across all samples showing comparatively high abundance of XRCC6 in all samples. Between nucleosome and chromatosome samples statistically significant relative differences were identified. B: Confirmation of enrichment by immunoblotting as described in Figure 4. C: Relative quantification of the band intensities of the XRCC6 immunoblot, performed with Image Lab.

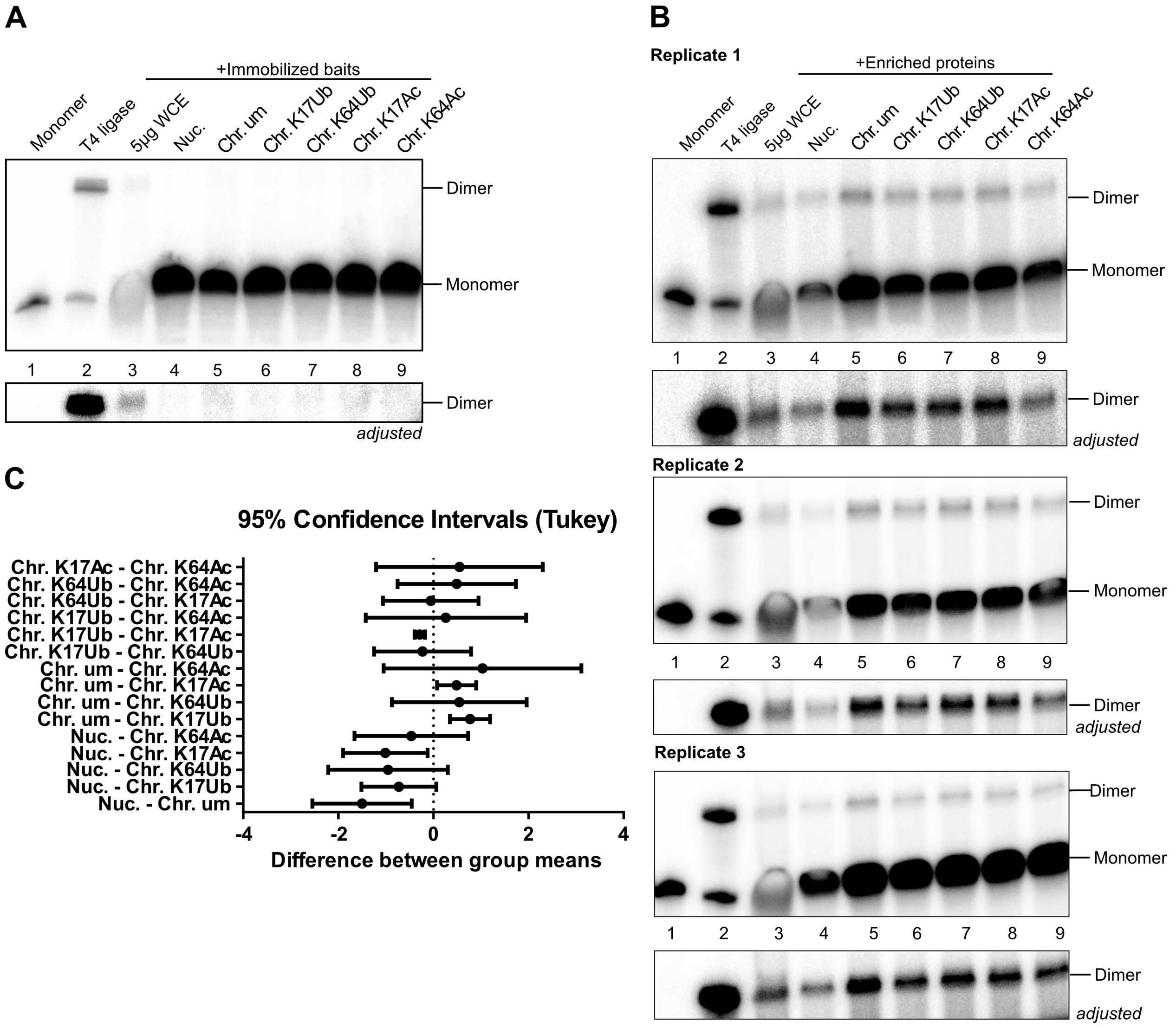

Figure S7: ***In vitro DSB* repair assay.** A: Autoradiography of denaturing PAGE analysis after incubation of a DSB mimic with either bait chromatosomes or nucleosome on beads does not show any formation of dimeric ligation product. B: Autoradiography of denaturing PAGE analysis after incubation of a DSB mimic with enriched proteins shows formation of dimeric ligation product is increased for intact unmodified chromatosomes and H1 variants relative to nucleosomes. The assay therefore functionally validates the binding of DSB repair proteins identified by AE-MS to intact chromatosomes. C: 95% confidence intervals of repeated measures one-way ANOVA with Tukey's multiple comparison test (confidence level 0.05) of evaluation of DSB repair assay triplicates.

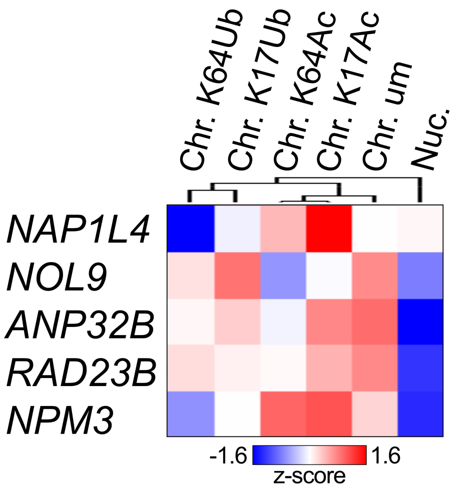

Figure S8: **Overlapping interactors of H1.2 and H1.2 in chromatosomes.** Interacting proteins both identified in interactomes of H1.2 on its own(1, 2) and in chromatosomes (this study). Shown is the mean z-score as found in this study.

Supplementary References

1. Höllmüller,E., Greiner,K., Kienle,S.M., Scheffner,M., Marx,A. and Stengel,F. (2021) Interactome of Site-Specifically Acetylated Linker Histone H1. J Proteome Res, **20**, 4443–4451.
2. Höllmüller,E., Geigges,S., Niedermeier,M.L., Kammer,K.-M., Kienle,S.M., Rösner,D., Scheffner,M., Marx,A. and Stengel,F. (2021) Site-specific ubiquitylation acts as a regulator of linker histone H1. Nat Commun, **12**, 3497.
